# Supplementary figures and images for: Comparative Proteomic Analysis of Secretory Proteins of Mycoplasma bovis and Mycoplasma mycoides subsp. mycoides Investigates Virulence and Discovers Important Diagnostic Biomarkers
Source: Vet Sci. 2023 Dec 1;10(12):685. doi: 10.3390/vetsci10120685 (PMC10748157; doi:10.3390/vetsci10120685)

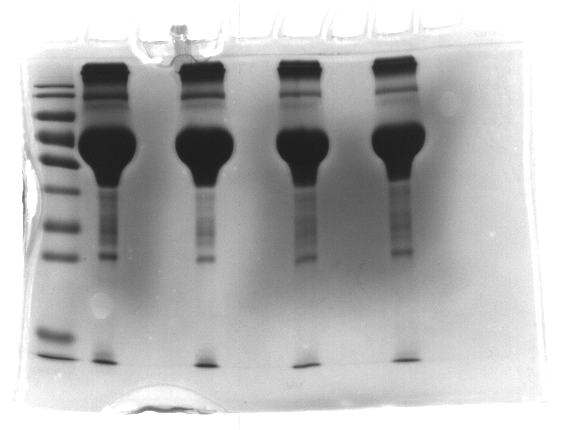

Supplement: Supplementary file 1 [file vetsci-10-00685-s001.zip › (S1)For-Fig.1C.Tif]

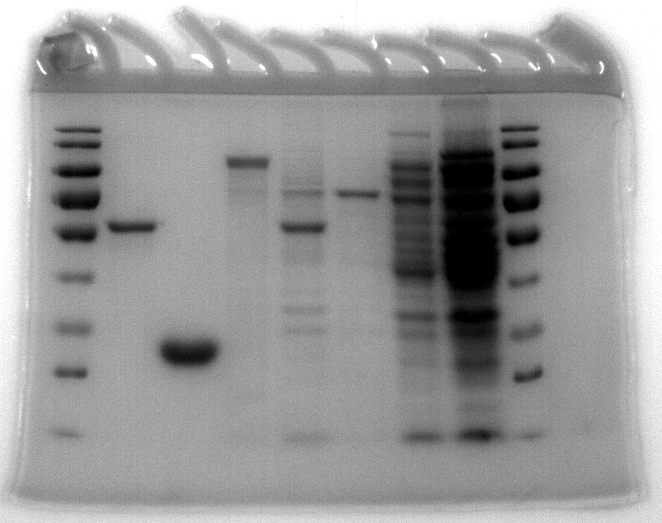

Supplement: Supplementary file 1 [file vetsci-10-00685-s001.zip › (S2) For-Fig.3A.Tif]

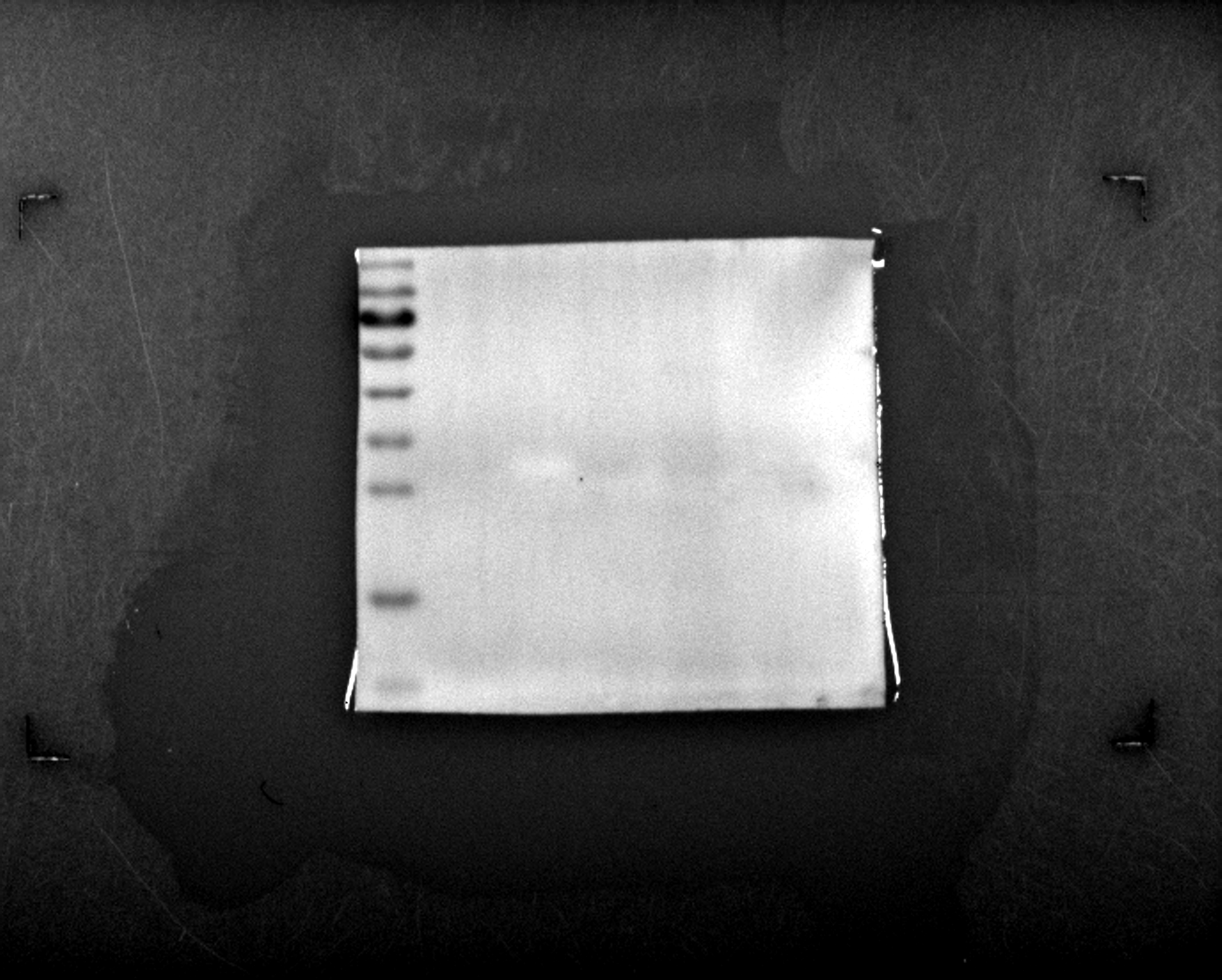

Supplement: Supplementary file 1 [file vetsci-10-00685-s001.zip › (S3) For-Fig.3B.Tif]

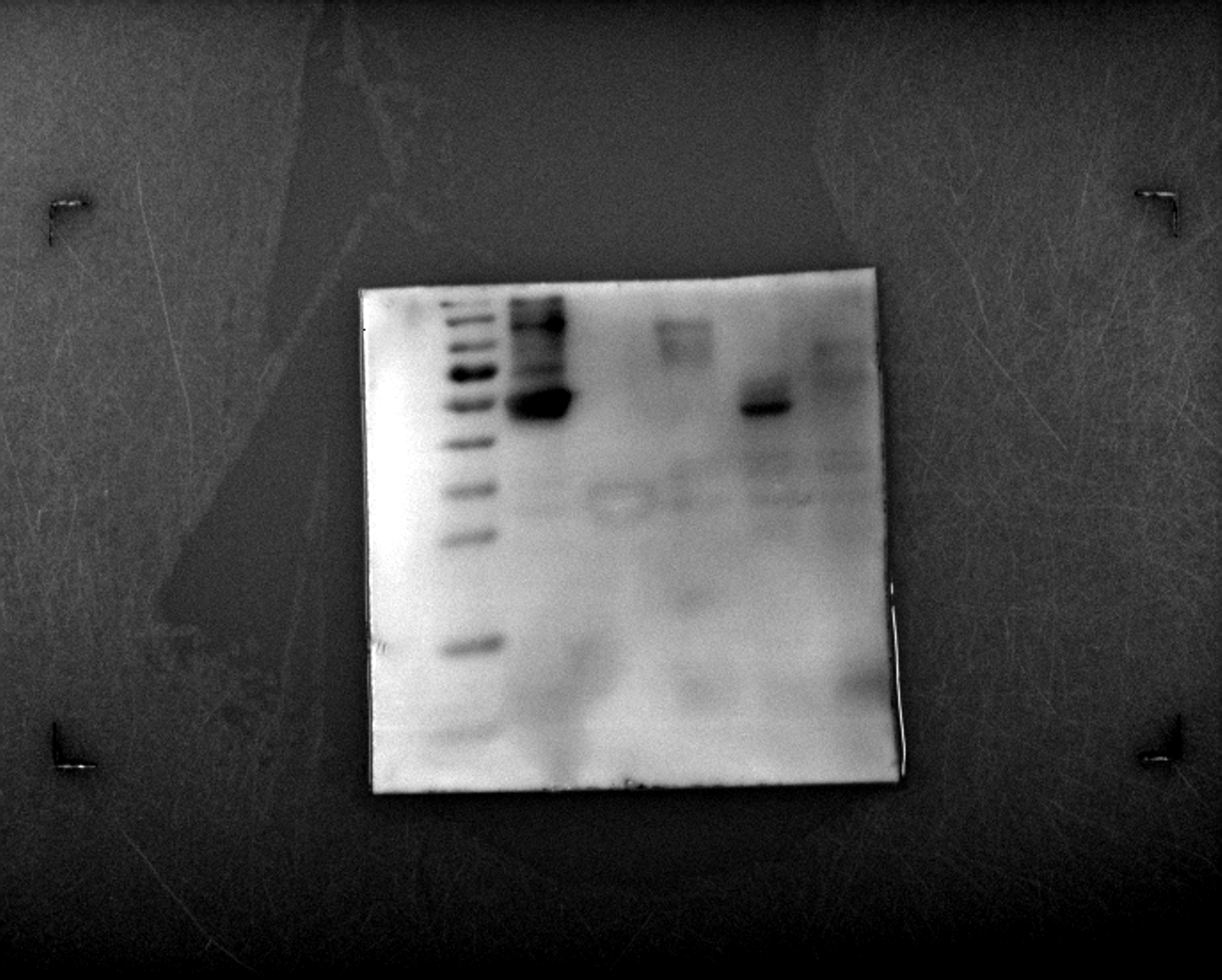

Supplement: Supplementary file 1 [file vetsci-10-00685-s001.zip › (S4) For-Fig.3C.Tif]

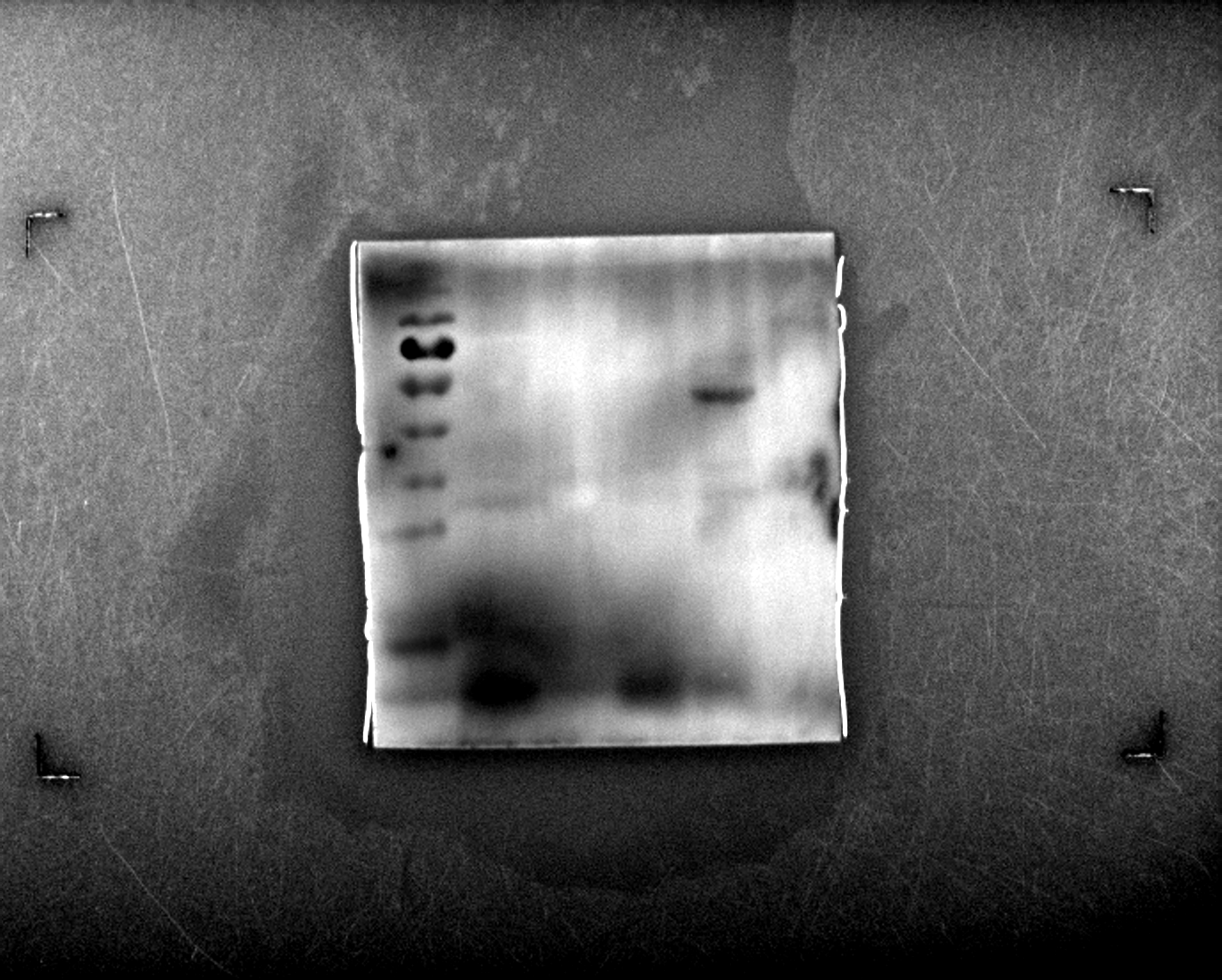

Supplement: Supplementary file 1 [file vetsci-10-00685-s001.zip › (S5) For-Fig.3D.Tif]

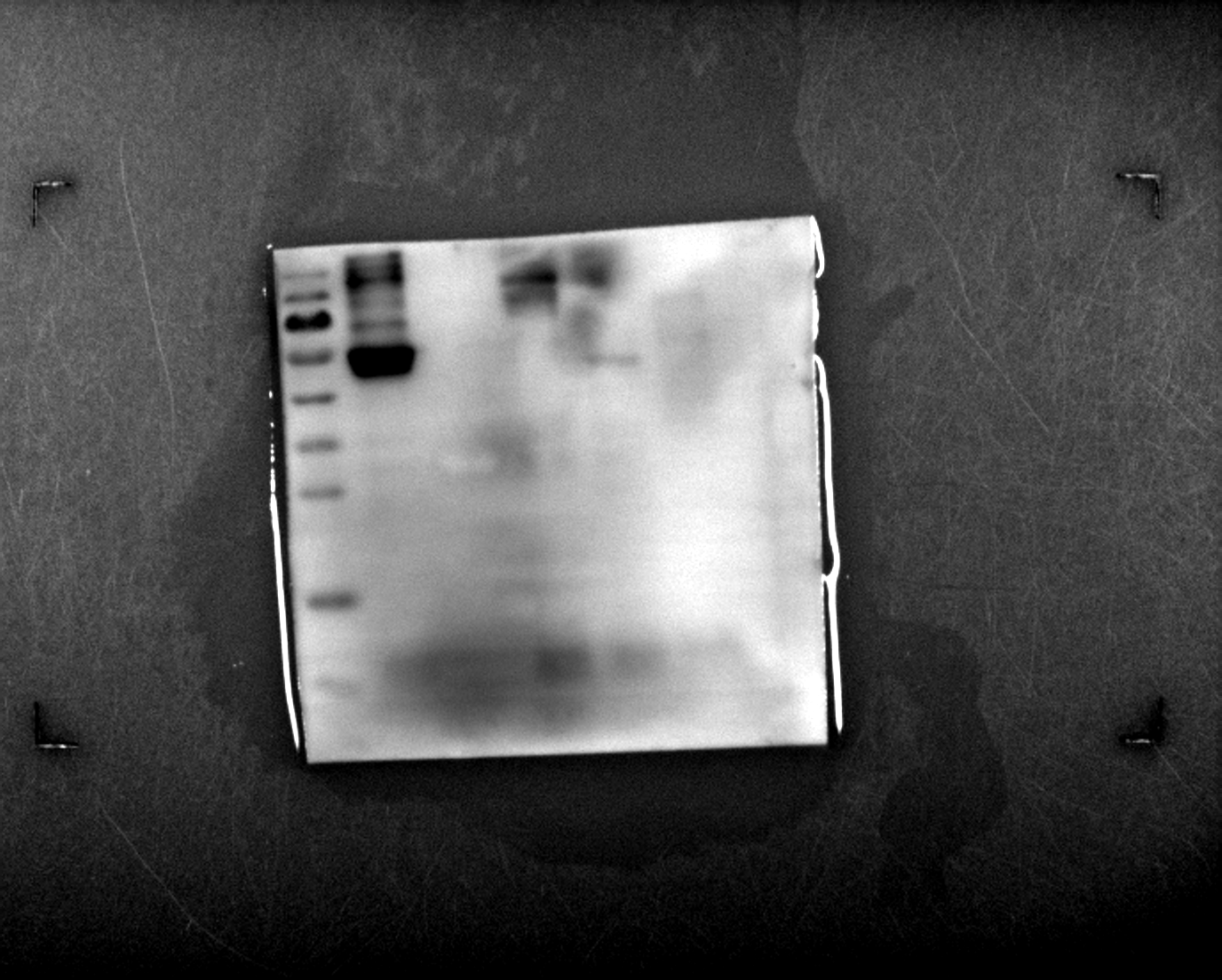

Supplement: Supplementary file 1 [file vetsci-10-00685-s001.zip › (S6) For-Fig.3E.Tif]

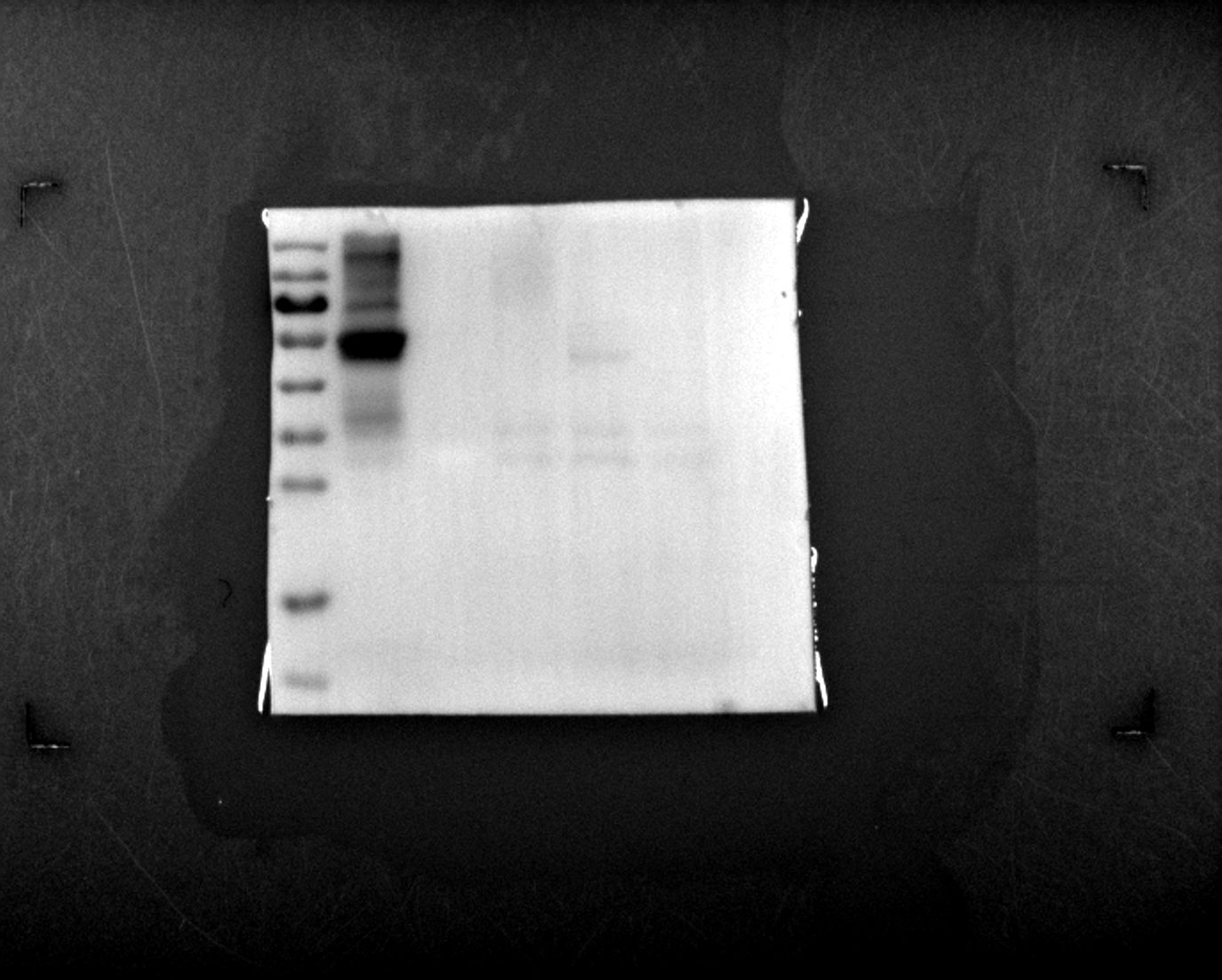

Supplement: Supplementary file 1 [file vetsci-10-00685-s001.zip › (S7) For-Fig.3F.Tif]

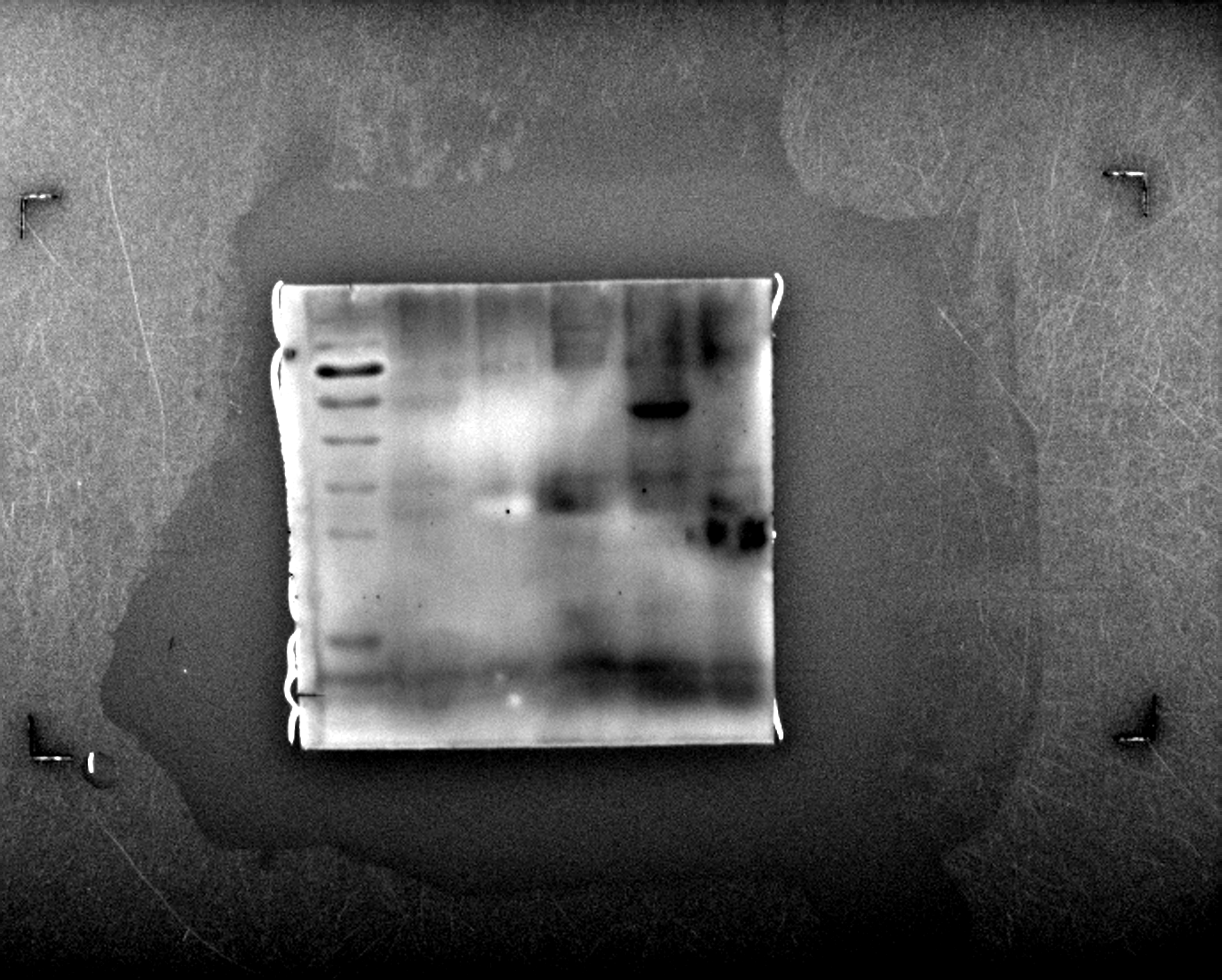

Supplement: Supplementary file 1 [file vetsci-10-00685-s001.zip › (S8) For-Fig.3G.Tif]
